# Activating Ionic Carbon Nitrides via Epitaxial Dyadic Coupling for Efficient Solar‐Driven H_2_O_2_ Generation

**DOI:** 10.1002/advs.77182

**Published:** 2026-08-15

**Authors:** Haijian Tong, Chenyan Zhao, Christian Mark Pelicano, Markus Antonietti

**Affiliations:** ^1^ Department of Colloid Chemistry Max Planck Institute of Colloids and Interfaces Potsdam Germany; ^2^ State Key Laboratory of Precision and Intelligent Chemistry University of Science and Technology of China Hefei Anhui China

**Keywords:** electron transfer, epitaxy, exciton, graphitic carbon nitride, heptazine, materials science, nanostructure, photocatalysis, quantum yield

## Abstract

Solar hydrogen peroxide synthesis is a sustainable alternative to the anthraquinone process, yet its efficiency is limited by charge separation and transport in polymeric photocatalysts. Ionic carbon nitrides like poly(heptazine imide) (PHI) offer tunable structures and electronic properties. Herein, we report an epitaxial strategy to construct a different *π*‐conjugated CN species (C_1_N_1_) on potassium poly(heptazine imide) (KPHI), forming a dyadic charge‐transfer nanostructure with strong interfacial coupling. Structural and spectroscopic analyses reveal that epitaxial registry at the interface induces orbital hybridization and a built‐in electric dipole, driving directional electron transfer from KPHI to C_1_N_1_ while retaining holes within the PHI. The conduction band of the composite shifts upward, providing a thermodynamic benefit for the oxygen reduction reaction (ORR). Theoretical calculations confirm the thermodynamic driving force for electron transfer from KPHI to C_1_N_1_ and strong interfacial interactions. The optimized composite achieves an AQY of 63.9% at 410 nm without cocatalysts, approaching the optimum for stirred powder reactors. In‐situ FTIR detects ∙O_2_^−^ and ∙OOH intermediates and further reveals interfacial water reconstruction with increased free water molecules that facilitate proton supply. Mechanistic studies confirm a two‐electron ORR pathway mediated by superoxide intermediates, promoted by the programmed charge‐transfer landscape.

## Graphical abstract

Epitaxial integration of a C_1_N_1_ phase onto KPHI creates a dyadic charge‐transfer nanostructure with strong interfacial electronic coupling. This architecture drives directional electron transfer from KPHI to C_1_N_1_, enabling efficient cocatalyst‐free solar H_2_O_2_ production.

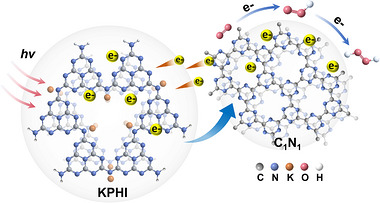

## 1 Introduction

Artificial photosynthesis is considered as a sustainable approach to harness solar energy for the sustainable production of value‐added, energy‐rich molecules [1]. Importantly, the ability to control charge carrier dynamics in semiconductors lies at the heart of photocatalysis and energy conversion [2]. While significant progress has been made in designing materials with high chemical stability, suitable band structures and light absorption properties, it is still important to improve how photogenerated electrons and holes are spatially separated, transported, and ultimately utilized [3, 4, 5].

Ionic carbon nitrides, such as poly(heptazine imide) (PHI), represent a unique class of polymeric semiconductors that bridge molecular and solid‐state systems [6, 7]. Their extended *π*‐conjugated frameworks with embedded ionic environments enable not only efficient light absorption but also the accumulation of photogenerated electrons, thereby facilitating multi‐electron redox chemistry [8]. However, the electronic structure of pristine PHI remains largely homogeneous, and introducing the ability to direct charge flow is therefore highly desirable [9]. The introduction of electronically coupled secondary phases that can act as charge‐transfer partners is a typical approach to realize better charge separation and direction [10, 11, 12, 13, 14, 15]. Covalent materials with extended *π*‐conjugation are particularly attractive in this regard, especially when stacked as 2D systems onto the PHI. Sensitization of semiconductors with carbon dots can be considered as a first step in this direction, but does not provide optimized electronic coupling [16, 17, 18].

Here, we present that epitaxial integration of a different carbon nitride phase onto ionic carbon nitrides can create a dyadic electronic architecture, in which interfacial orbital hybridization gives rise to new charge‐transfer (CT) states. This special dyadic configuration enables strong electronic coupling and spatially resolved charge separation, allowing electrons and holes to be selectively localized in distinct domains [19]. We realize this concept through the synthesis of a C_1_N_1_‐KPHI hybrid via condensation in salt melts, where guanine‐derived C_1_N_1_ domains grow epitaxially on KPHI. Structural and spectroscopic analyses reveal the formation of a coherent interface that promotes charge‐transfer‐type orbital hybridization. Band structure analysis shows that the conduction band of the composite shifts upward to a more negative position, which is thermodynamically favorable for the ORR process. DFT calculations confirm that the Fermi level of KPHI (−4.51 eV) is higher than that of C_1_N_1_ (−5.41 eV), providing the thermodynamic driving force for electron transfer from KPHI to C_1_N_1_, while density of states (DOS) analysis further confirms strong interfacial interactions. Photoexcited electrons are preferentially transferred from KPHI to C_1_N_1_, where they are stabilized and accumulated, while holes remain confined within the KPHI framework. This dyadic architecture suppresses recombination and enables directional charge transport across the interface. As a model reaction, photocatalytic H_2_O_2_ generation via oxygen reduction was analyzed, and high efficiencies exceeding 60% apparent quantum yield could be realized. The in‐situ FTIR spectroscopy not only detects key reaction intermediates (∙O_2_^−^ and ∙OOH), confirming the superoxide‐mediated two‐electron ORR pathway, but also reveals interfacial water reconstruction with an increase in free water molecules, which enhances proton transfer kinetics. These findings offer direct evidence for the reaction mechanism and highlight the crucial role of interfacial water dynamics in promoting H_2_O_2_ generation. More broadly, this work establishes epitaxial dyadic interface engineering as a strategy to program the electronic structure of polymeric semiconductors, moving beyond conventional heterojunction design toward adaptive and functionally integrated materials.

## 2 Results and Discussion

### 2.1 Materials Characterization

**FIGURE 1:**
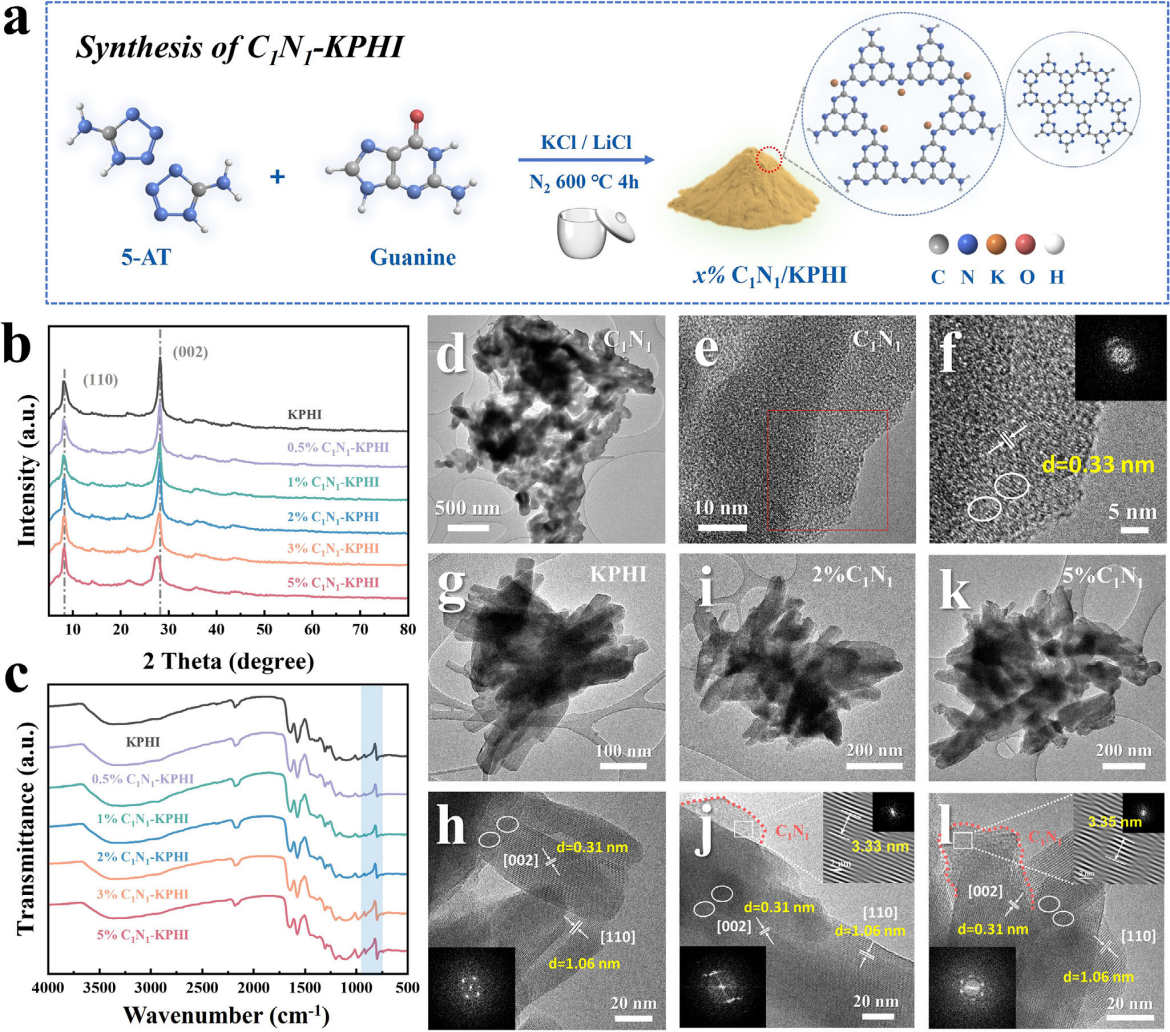
(a) Illustration for the synthesis process of *x*%C_1_N_1_‐KPHI with proposed structures. (b) XRD patterns and (c) FTIR spectra of KPHI and all *x*%C_1_N_1_‐KPHI. HR‐TEM images of (d,e) C_1_N_1_, with (f) enlarged area pattern, (g,h) KPHI, (i,j) 2%C_1_N_1_‐KPHI and (k,l) 5%C_1_N_1_‐KPHI.

The catalysts were synthesized via a straightforward one‐step thermal condensation approach, as illustrated in Figure 1a. Typically, 2.5 g of 5‐aminotetrazole was mixed with 12.5 g of a KCl/LiCl eutectic salt mixture (mass ratio 0.55:0.45), followed by the addition of a specific amount of guanine, which served as a C_1_N_1_ precursor. Interestingly, the two monomers do not react with each other but instead condense into separate phases. The mixture was then ball‐milled at 25 Hz for 5 min, transferred to a crucible, and thermally annealed at 600 °C for 4 h under a nitrogen atmosphere. The obtained yellowish‐brown powders were denoted as *x*%C_1_N_1_‐KPHI, where *x* indicates the weight percentage of guanine relative to 5‐aminotetrazole (5‐AT). For comparison, pristine KPHI and C_1_N_1_ were also synthesized under identical conditions in the absence of guanine and 5‐AT, respectively. The structural and chemical properties of the synthesized photocatalysts were characterized by x‐ray diffraction (XRD) and Fourier transform infrared (FTIR) spectroscopy. In the XRD analysis, pure KPHI exhibits two characteristic peaks at 8.1° and 28.1°, which correspond to the (110) and (002) crystal planes, respectively (Figure S1a) [20]. In comparison, C_1_N_1_ displays a single broad peak centered around 27.1°, assigned to the (002) crystal plane [21]. These distinct diffraction patterns confirm the successful synthesis of both KPHI and C_1_N_1_ structures. FTIR spectroscopy provides further insight into the structure of C_1_N_1_ (Figure S1b). The disappearance of characteristic vibration peaks from the guanine precursor following thermal treatment confirms its transformation into C_1_N_1_. A broad absorption band in the 1200–1600 cm^−1^ range is attributed to stretching vibrations of C‐N heterocycles, indicative of aromatic nitrogen‐rich structures. Another broad band between 2800 and 3700 cm^−1^ corresponds to N─H stretching and bending modes and/or physically adsorbed water, suggesting that condensation of guanine‐derived intermediates may be incomplete. Additionally, peaks at 1250 and 1582 cm^−1^ signify the presence of C═C and C═N functional groups, consistent with partial graphitization and the formation of heteroaromatic frameworks [22].

The Raman spectrum of C_1_N_1_ shows a peak centered at 1001 cm^−1^, which is assigned to the vibrational mode of the triazine ring (Figure S1c). Two main peaks at approximately 1354 and 1502 cm^−1^ correspond to overlapping C─N and C─C bond vibrations within the triazine framework. Additionally, the peak at 2798 cm^−1^ indicates higher‐order vibrations typical of stacked carbonaceous layers [23]. Thermogravimetric analysis (TGA) assesses the thermal stability of the starting materials. As shown in Figure S1d, 5‐AT, used for KPHI synthesis, decomposes around 200°C, whereas guanine decomposes at a higher temperature (∼ 450°C). This disparity suggests that the poly(heptazine) network likely occurs before C_1_N_1_ formation when both precursors are combined, which is advantageous as it minimizes the likelihood of C_1_N_1_ interfering with KPHI assembly. This proposed sequential formation is further supported by XRD data (Figure 1b), where all *x*%C_1_N_1_‐KPHI hybrid samples exhibit the characteristic diffraction peaks of KPHI, indicating that its core structure remains largely intact after incorporating C_1_N_1_. A minor shift of these peaks toward lower angles hints at possible interactions or integration between KPHI and C_1_N_1_. In Figure 1c, KPHI and its hybrid composites exhibit nearly identical vibrational features, reinforcing the structural integrity of KPHI upon C_1_N_1_ incorporation. A distinct peak at 801 cm^−1^ corresponds to the out‐of‐plane breathing vibration of the heptazine ring, while vibration at 910 and 987 cm^−1^ is linked to C─N bonds associated with K^+^ coordination to ─NC_2_ units. The range between 1200 and 1700 cm^−1^ contains multiple bands attributed to stretching and bending vibrations of heptazine derivatives. Broad absorption in the 3000–3600 cm^−1^ range is attributed to terminal amino (─NH_2_) groups and adsorbed water [24, 25]. Notably, the intensity of this broad band diminishes progressively with increasing C_1_N_1_ content, implying that C_1_N_1_ may react or interact with the terminal amino groups during hybrid formation.

Scanning electron microscopy (SEM) and transmission electron microscopy (TEM) analyses revealed the morphological evolution of the photocatalysts. As illustrated in Figure S2, pure C_1_N_1_ appeared as small fragments or oval‐shaped particles, while pure KPHI displayed a distinct rod‐like structure. Upon the formation of the hybrid C_1_N_1_‐KPHI materials, the nanorod architecture of KPHI was generally preserved. However, increasing amounts of fine particles were observed decorating the nanorods as the C_1_N_1_ content increased. Especially, the 10% and 20%C_1_N_1_‐KPHI catalysts showed extensive surface coverage by C_1_N_1_ particles. This increased surface coverage is likely responsible for the reduced crystallinity observed, in agreement with the XRD analysis. High‐resolution TEM imaging reveals that C_1_N_1_ possesses a fragmented or pebble‐like morphology, with magnified selected‐area images displaying a clear lattice spacing of 0.33 nm, corresponding to the (002) crystallographic planes (Figure 1d–f). Elemental mapping images confirm the uniform distribution of C, N, and K within the C_1_N_1_ structure, verifying successful synthesis (Figure S3). For the heterojunction systems, TEM images of pristine KPHI and the C_1_N_1_‐KPHI composites consistently display a rod‐like morphology. Notably, smaller fragments are observed surrounding the nanorods, and their presence becomes more pronounced with increasing C_1_N_1_ content, as illustrated in Figure S4. Further high‐resolution TEM analysis provides compelling evidence for the formation of a heterojunction between C_1_N_1_ and KPHI (Figure 1g–l). In comparison to pure KPHI, both 2%C_1_N_1_‐KPHI and 5%C_1_N_1_‐KPHI catalysts exhibit distinct lattice spacings of 1.06 and 0.31 nm, which correspond to the (110) and (002) planes, respectively. The regions marked with red dots in Figure 1j,l correspond to C_1_N_1_‐enriched domains. Notably, C_1_N_1_ appears to grow epitaxially along KPHI surfaces, exhibiting a lattice spacing of 0.33 nm that is consistent with the (002) planes of C_1_N_1_. The epitaxial growth is presumably driven by lattice matching along the stacking direction together with interfacial electron transfer. As the C_1_N_1_ content increases, more C_1_N_1_ domains are observed, confirming enhanced surface coverage in higher‐loaded samples. Elemental mapping provides additional confirmation of the homogeneous distribution of K, C, and N in both KPHI and 2%C_1_N_1_‐KPHI hybrid (Figure S5a,b). Quantitative elemental analysis reveals a C:N atomic ratio of approximately 1:1 in C_1_N_1_, and all composite samples show an increased carbon and nitrogen contents following C_1_N_1_ incorporation (Table S1). Together, these findings confirm the successful synthesis of C_1_N_1_ and its effective integration into a heterojunction structure with KPHI.

All composite samples exhibit increased specific surface areas compared to pristine KPHI, a result attributed to the inclusion of C_1_N_1_, which itself has a moderately higher surface area of 79 m^2^ g^−1^, as shown in Figure S6 and Table S2. This enhanced surface area is beneficial for photocatalytic processes, as it provides more active sites for reactant adsorption, thereby improving reaction kinetics and overall catalytic performance. To further examine the chemical structure and elemental composition, x‐ray photoelectron spectroscopy (XPS) was employed. The XPS survey spectra verified the presence of C, N, K, and O elements in all synthesized catalysts, with no significant elemental impurities detected (Figures S7a and S8a). For the pristine C_1_N_1_ material, the high‐resolution C 1s XPS spectrum revealed three distinct carbon environments with binding energies at 284.7 eV (C═C), 285.9 eV (N═C(C) ─N), and 287.2 eV (N─ (N)C═N) (Figure S7b). Furthermore, the N 1s spectrum was deconvoluted into three main peaks at 398.1 eV (N‐pyridinic), 399.7 eV (N‐quaternary), and 401 eV (─NH_x_) (Figure S7c) [26]. To further elucidate the chemical structure of the hybrid C_1_N_1_‐KPHI catalysts, detailed high‐resolution XPS analyses were performed on all composite samples. The C 1s high‐resolution spectra for KPHI displayed characteristic peaks at 284.8 eV (C─C), 286.5 eV (C‐NH_x_), and 288.16 eV (N─C═N) (Figure S8b) [27]. Across the composite series, a gradual decrease in the intensity of the 286.5 eV peak was observed, which correlates with a reduction in terminal ‐NH_x_ groups, as also supported by FTIR data. This attenuation does not stem from changes in cyano groups, whose spectral features remain unaltered. In the C_1_N_1_‐KPHI hybrids, a slight shift in the C 1s binding energies was detected relative to pristine KPHI, likely due to electronic interactions induced by the adjacent C_1_N_1_ phase. Notably, the N‐C = N peak shifted negatively by 0.09 eV in the 2%C_1_N_1_‐KPHI sample, suggesting an electron transfer between C_1_N_1_ and KPHI [28]. The N 1s high‐resolution spectra revealed four distinct components at 398.5 eV (sp^2^‐hybridized N in C─N═C), 399.8 eV (tertiary N, (N‐C_3_), 400.9 eV (terminal ─NH_x_ or ─C≡N groups), and 403.5 eV (surface oxidized nitrogen) (Figure S8c) [29]. Additionally, a negative shift of 0.07 eV in the N═C─N peak of the N 1s spectra parallels the shift observed in the C 1s spectra, further reinforcing the evidence for electronic and chemical interactions between the two phases. This shift supports the occurrence of electron transfer from KPHI to C_1_N_1_, likely driven by the built‐in electric field at the heterojunction interface [30]. Furthermore, Table S3 reveals a decrease in the N_2_C/N_3_C ratio with increasing C_1_N_1_ content, suggesting the formation of nitrogen vacancies within the structure. To explore the role of these structural defects, electron paramagnetic resonance (EPR) spectroscopy was conducted on both pristine KPHI and the 2%C_1_N_1_‐KPHI hybrid (Figure S9). In both cases, the EPR spectra exhibited a primary Lorentzian signal with a g‐value of 2.003, characteristic of unpaired electrons localized on sp^2^‐hybridized nitrogen atoms in the *π*‐conjugated carbon nitride framework. The markedly stronger EPR signal in the 2%C_1_N_1_‐KPHI catalyst suggests a higher concentration of unpaired electrons [31]. This observation provides additional confirmation that structural defects are effectively introduced through hybridization with C_1_N_1_, thereby facilitating photoinduced charge transfer, which is advantageous for photoactivity.

### 2.2 Photocatalytic Performance

**FIGURE 2:**
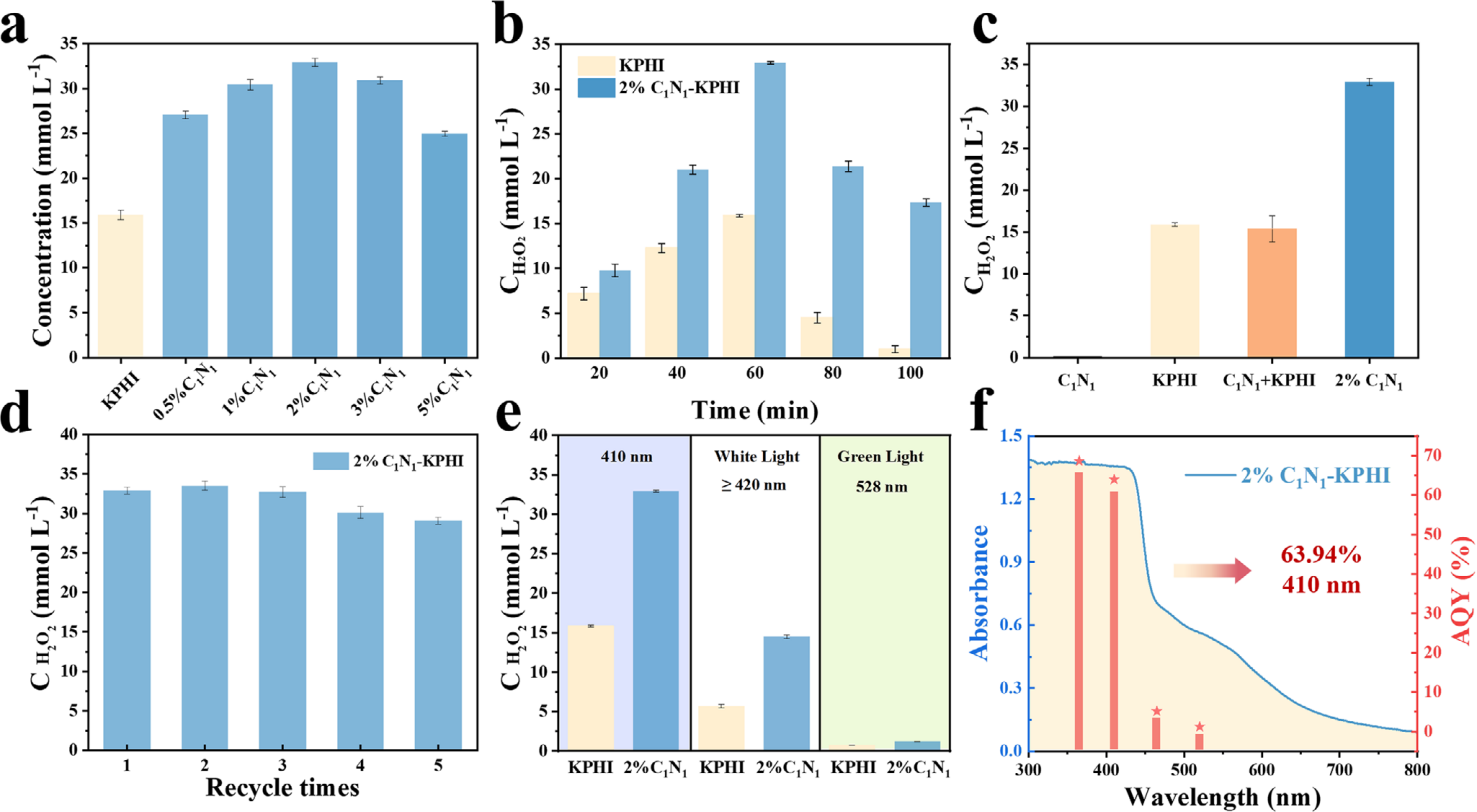
(a) Influence of C_1_N_1_ loading on the H_2_O_2_ evolution performance of KPHI. (b) Different reaction time of KPHI and 2%C_1_N_1_‐KPHI. (c) H_2_O_2_ evolution performance of C_1_N_1_, KPHI, C_1_N_1_ and KPHI (mixed by grinding), 2%C_1_N_1_‐KPHI. (d) Recyclability tests for 2%C_1_N_1_‐KPHI in H_2_O_2_ production. (e) Photocatalytic H_2_O_2_ evolution activities of KPHI and 2%C_1_N_1_‐KPHI using different excitation wavelengths, and (f) UV–vis spectrum and AQY values for 2%C_1_N_1_‐KPHI as a function of the irradiation wavelength.

The photocatalytic performance of the synthesized materials for H_2_O_2_ generation was systematically evaluated under 410 nm LED irradiation, in the absence of a cocatalyst (Figure 2a). For evaluation, each catalyst (2.5 mg mL^−1^) was suspended in an oxygen‐saturated aqueous glycerol solution (10% w/w). Among the tested samples, the 2%C_1_N_1_‐KPHI composite exhibited the highest activity, generating 32 mM of H_2_O_2_ after 1 h — a yield twice that of pristine unmodified KPHI (16 mM). Notably, glycerol oxidation produced glyceraldehyde (60 mM) as the major product, together with glycolic acid (20 mM), dihydroxyacetone (8.5 mM), and formic acid (5 mM) (Figure S10) [32, 33]. These results demonstrate that coupling photocatalytic H_2_O_2_ production with selective glycerol oxidation enables the simultaneous generation of value‐added oxygenates. A comprehensive investigation of the oxidation kinetics and the separation of the oxidation products from H_2_O_2_ will be pursued in future work.

Further increases in C_1_N_1_ content beyond 2 wt.% led to a gradual decline in photocatalytic performance, though still surpassing the activity of KPHI alone. This decline is attributed to excessive C_1_N_1_ loading, which causes local aggregation (as observed by SEM in Figure S2) and reduced crystallinity (broader and weaker XRD peaks in Figure 1b). These structural changes negatively affect charge transfer and reduce the intrinsic active sites of KPHI, ultimately resulting in decreased activity. Furthermore, a decline in H_2_O_2_ concentration after 1 h was observed for both pristine KPHI and 2%C_1_N_1_‐KPHI, which is attributed to the intrinsic H_2_O_2_ decomposition ability of the materials themselves under prolonged irradiation (Figure 2b). To further verify this, we performed H_2_O_2_ decomposition experiments under identical conditions. After 1 h of reaction, pristine KPHI decomposed 98.7% of the initially added H_2_O_2_, while the 2%C_1_N_1_‐KPHI composite decomposed only 57.6%, confirming that the composite effectively suppresses H_2_O_2_ decomposition (Figure S11). As a result, while the apparent activity of KPHI dropped to nearly zero after 100 min (i.e. the rates of H_2_O_2_ formation and decomposition reach a dynamic equilibrium), the 2%C_1_N_1_‐KPHI still retained considerable activity, producing 17 mM of H_2_O_2_. This further demonstrated that the epitaxially coupled C_1_N_1_‐KPHI catalyst effectively suppresses the undesired H_2_O_2_ decomposition pathway and maintains superior photocatalytic performance compared with KPHI during prolonged irradiation. To better understand the role of C_1_N_1_ in the composites, pristine C_1_N_1_ and a physical mixture of KPHI with 2%C_1_N_1_ (prepared by manual grinding, labeled as 2%C_1_N_1_‐KPHI‐M) were also tested (Figure 2c). Pristine C_1_N_1_ exhibited negligible photocatalytic activity, consistent with its intrinsic nature as a carbonaceous material lacking photocatalytic ability. For the physically mixed sample, UV–vis absorption spectrum in Figure S12 remained essentially unchanged compared with pristine KPHI, and the photocatalytic activity showed no significant enhancement. These results demonstrate that mere physical blending does not enable effective charge separation via directional electron transfer.

The improved performance of the 2%C_1_N_1_‐KPHI composite points to the formation of the built‐in electric CT field, which facilitated efficient electron transfer to O_2_, driving the reduction reaction. 2%C_1_N_1_‐KPHI also demonstrated outstanding stability and recyclability. As shown in Figure 2d, the H_2_O_2_ concentration showed only a slight decrease over five consecutive reaction cycles, which we attribute to the minor catalyst loss during recovery between runs. Moreover, the main diffraction peaks of the recovered catalyst were well preserved in the XRD pattern (Figure S13), confirming the structural integrity of the material throughout the photocatalytic reaction. To evaluate the wavelength‐dependent photocatalytic behavior of 2%C_1_N_1_‐KPHI relative to KPHI, H_2_O_2_ generation tests were performed under various diode light sources (Figure 2e). The 2%C_1_N_1_‐KPHI catalyst exhibited a marked improvement in activity under simulated white light (*λ* ≥ 420 nm), delivering an activity 1.54 times higher than that of pristine KPHI. Under green LED illumination (*λ* = 528 nm), it also showed superior performance, reaching an activity 0.64 times higher than KPHI, with the less pronounced enhancement at longer wavelengths arising from the lower photon flux density of the green light source. These enhancements, particularly evident at longer wavelengths, suggest that the heterojunction between C_1_N_1_ and KPHI adds a further charge‐transfer band which effectively broadens the light absorption range of the photocatalyst. Furthermore, the apparent quantum yields (AQY) of 2%C_1_N_1_‐KPHI were determined to be 68.62% at 365 nm and 63.94% at 410 nm (Figure 2f), about two times higher than that of pristine KPHI (AQY_410nm_ = 30%). The performance achieved here outperforms most previously reported carbon nitride‐based photocatalysts (Table S4). However, we also want to point out that deviations from 100% AQY are mostly due to imperfect reactor engineering, as indeed stirred powder reactors show incomplete light absorption due to light transmission, light scattering and imperfect light focus [34, 35, 36], which make it very difficult to ensure that every photon is absorbed by the photocatalyst.

### 2.3 Optical and Electrochemical Characterization

**FIGURE 3:**
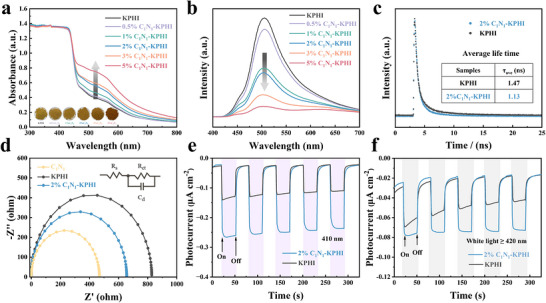
(a) UV–vis DRS spectra of samples (inset: optical images of KPHI and *x*%C_1_N_1_‐KPHI powders). (b) Room temperature steady‐state PL emission spectra of all samples. (c) Solid‐state time‐resolved PL decay of KPHI and 2%C_1_N_1_‐KPHI. (d) Electrochemical impedance spectroscopy Nyquist plots of C_1_N_1_, KPHI and 2%C_1_N_1_‐KPHI. Transient photocurrent (e) *λ* = 410 nm and (f) white light for KPHI and 2%C_1_N_1_‐KPHI in 0.2 M Na_2_SO_4_ aqueous solution.

To unravel the underlying reasons for the altered photocatalytic performance of the 2%C_1_N_1_‐KPHI hybrid, an extensive set of optical and photo(electro)chemical analyses was conducted. UV–vis diffuse reflectance spectroscopy (DRS) served as the primary method for initial optical assessment. As illustrated in Figure S14a, pure C_1_N_1_ exhibits broad spectral absorption, consistent with its black appearance and indicative of a well‐developed, graphite‐like *π*‐conjugated system [37]. In contrast, pristine KPHI shows pronounced absorption in the 300–450 nm range due to *π* → *π* ^*^ transitions, along with a secondary broad band in the 450–650 nm range, attributed to n → *π* ^*^ transitions enabled by K^+^ ions, which help reduce the molecular symmetry constraints [38]. Upon formation of the hybrid material, a visible color transition from yellow to yellowish‐brown is noted, accompanied by a red shift in the absorption edge that increases with higher C_1_N_1_ content (Figure 3a and Figure S14b). This shift is mainly ascribed to partial charge transfer involving the upper electrons of the PHI valence band, implying enhanced solar light absorption and a larger potential for photocarrier generation. To examine the charge separation and transfer processes in the 2%C_1_N_1_‐KPHI catalyst, a series of characterizations were conducted, including steady‐state photoluminescence (PL), time‐resolved PL (TRPL), electrochemical impedance spectroscopy (EIS) and transient photocurrent response (TPR). Steady‐state PL spectra reveal that pristine KPHI exhibits a strong emission peak, reflecting substantial radiative recombination of charge carriers (Figure 3b). Upon the addition of C_1_N_1_, the PL intensity notably declines, indicating suppressed charge recombination [39]. This phenomenon can be attributed to the electron transfer from KPHI to C_1_N_1_. TRPL analysis offers additional insight, showing a decrease in the average carrier lifetime from 1.47 ns for pristine KPHI to 1.13 ns for 2%C_1_N_1_‐KPHI (Figure 3c). This shorter lifetime reflects faster charge extraction, ultimately improving photocatalytic activity. As reveled by impedance analysis in Figure 3d, the smaller semicircle observed for C_1_N_1_‐modified PHI quantifies the lower charge transfer resistance compared with KPHI [40]. These improvements in charge separation and conductivity are further corroborated by TPR measurements (Figure 3e,f). The 2%C_1_N_1_‐KPHI composite demonstrates a significantly higher and more stable photocurrent than KPHI alone. Under 410 nm irradiation, its photocurrent is 2.5 times higher than that of pristine KPHI, and about 1.7 times higher under low‐intensity white light.

### 2.4 Mechanistic Investigation

To gain deeper insight into the dominant reaction mechanism within the synthesized composite, a series of reactive species quenching experiments were carried out. Furthermore, the reaction kinetics were systematically investigated by linear sweep voltammetry (LSV) using a rotating disk electrode (RDE), enabling detailed analysis of the electron transfer dynamics during photocatalytic H_2_O_2_ production. As shown in Figure S15, no H_2_O_2_ was detected either in the absence of light or without the catalyst, confirming the role of photocatalysis in the process. The requirement for oxygen was also clearly established: when the reaction system was purged with N_2_, H_2_O_2_ production was minimal, while exposure to air resulted in substantial H_2_O_2_ production. In this system, H_2_O_2_ primarily proceeds through the reduction of molecular O_2_ by photogenerated electrons. This process typically proceeds via two main pathways: a sequential two‐electron reduction (O_2_ → ∙O_2_^−^ → H_2_O_2_) or a direct two‐electron reduction (O_2_ → H_2_O_2_) [41].

**FIGURE 4:**
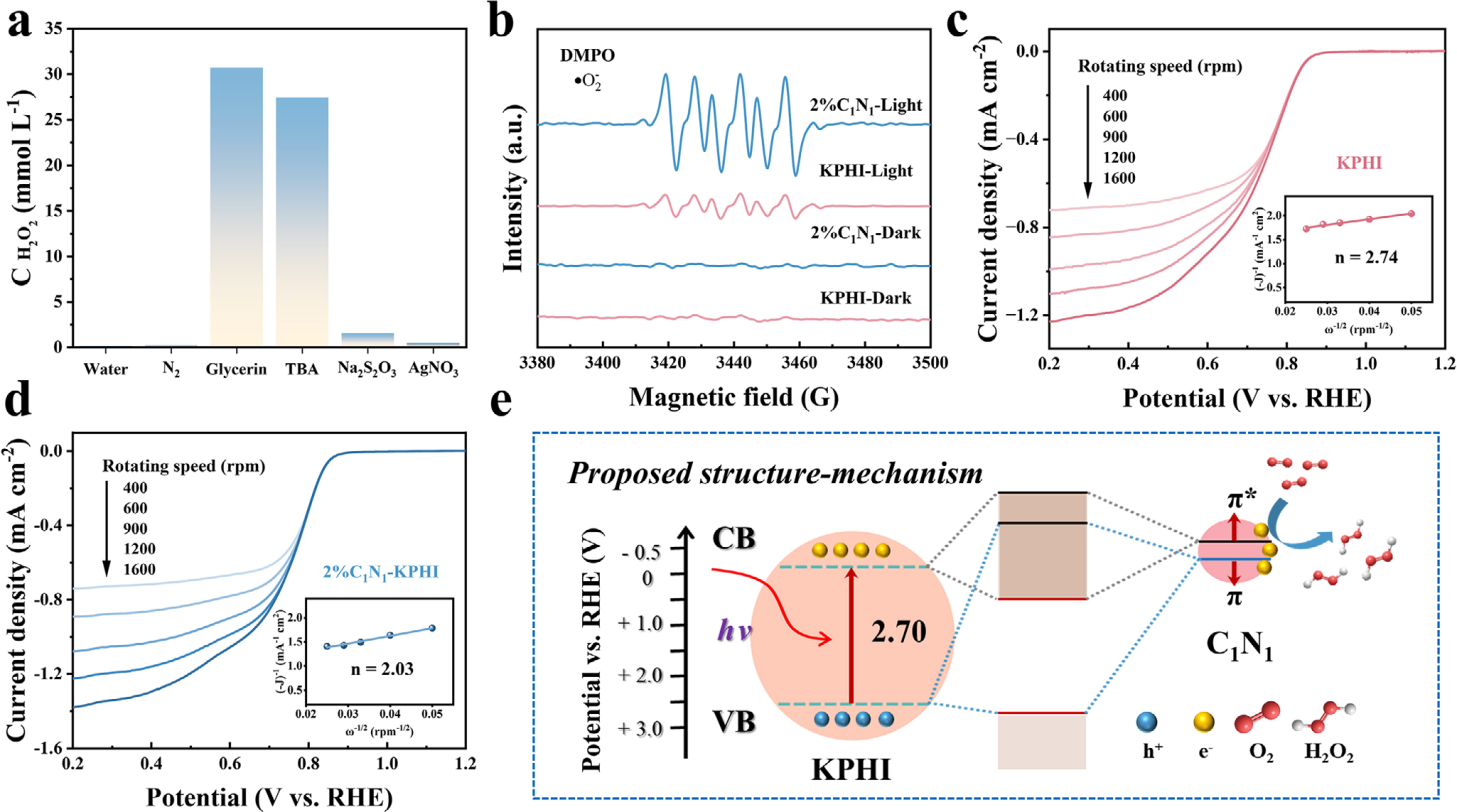
(a) Influence of different scavengers on the photocatalytic H_2_O_2_ production of 2%C_1_N_1_‐KPHI, (b) EPR spectra of DMPO‐∙O_2_^−^ over KPHI and 2%C_1_N_1_‐KPHI, and LSV curves of (c) KPHI and (d) 2%C_1_N_1_‐KPHI tested by RDE (Inset: fitted K‐L plot). (e) proposed dyadic structure formed via charge‐transfer‐type orbital hybridizations at the KPHI and C_1_N_1_ interface.

To identify the active intermediates involved in the reaction, radical scavenging experiments were conducted utilizing AgNO_3_ (e^−^), tert‐butyl alcohol (TBA, ∙OH), and sodium thiosulfate (Na_2_S_2_O_3_, ∙O_2_^−^), as shown in Figure 4a [42, 43]. A marked decrease in H_2_O_2_ production upon addition of AgNO_3_ highlighted the critical role of photogenerated electrons. TBA caused only a partial reduction in yield, indicating that ∙OH radicals are not the primary contributors to H_2_O_2_ generation. This conclusion is further supported by the continued detection of H_2_O_2_ in systems lacking glycerin or under nitrogen‐purged conditions. The presence of ∙OH radicals was nevertheless confirmed by EPR spectroscopy following light irradiation of the 2%C_1_N_1_‐KPHI catalyst (Figure S16), which implies that this channel exists as a side reaction. More importantly, the introduction of Na_2_S_2_O_3_ led to an almost complete suppression of H_2_O_2_ formation, conclusively demonstrating that ∙O_2_^−^ radicals are key intermediates in the reaction. These findings collectively suggest that the H_2_O_2_ generation process in the 2%C_1_N_1_‐KPHI system proceeds via two consecutive one‐electron reduction steps. This conclusion is directly supported by EPR spectroscopy, which showed no ∙O_2_^−^ radicals in the dark, but a clear signal under 410 nm light exposure, with a significantly stronger response from 2%C_1_N_1_‐KPHI compared to pristine KPHI (Figure 4b). This indicates that the heterojunction facilitates more efficient O_2_ to ∙O_2_^−^ conversion, thereby boosting H_2_O_2_ production. To further probe the ORR kinetics and verify the proposed mechanism, RDE measurements were performed for both catalysts. In the same potential range, 2%C_1_N_1_‐KPHI exhibited higher cathodic current densities, signifying superior ORR kinetics (Figure 4c,d). Analysis using Koutecky–Levich (K‐L) plots revealed electron transfer numbers of 2.74 for KPHI and 2.03 for 2%C_1_N_1_‐KPHI. The value of 2.74 for pristine KPHI suggests that the oxygen reduction reaction proceeds via a mixed pathway, involving both a two‐electron reduction to H_2_O_2_ and a four‐electron reduction to H_2_O, resulting in an apparent electron transfer number approaching 3. In contrast, the average value for 2%C_1_N_1_‐KPHI, being close to 2, strongly supports the predominance of a two‐electron reduction pathway leading to H_2_O_2_ formation, rather than the four‐electron pathway yielding water. The decrease in the K–L‐derived average electron transfer number from ∼3 for pristine KPHI to ∼2 for 2%C_1_N_1_‐KPHI reveals a significant mechanistic shift in the ORR. The construction of the dyadic structure suppresses the desorption of the ∙O_2_^−^ intermediates, confining them to the surface and steering the reaction toward a 2e^−^ pathway for H_2_O_2_ production [44]. Based on all experimental data, we propose that the synergistic dyadic structure composed of KPHI (with a bandgap of approximately 2.7 eV) and C_1_N_1_ facilitates the formation of optically active charge‐transfer transitions (Figure 4e) [17, 18]. In this configuration, photoexcited electrons are preferentially transferred to charge‐transfer states predominantly localized on C_1_N_1_, while the corresponding holes remain electronically and spatially associated with the KPHI domains. This spatial charge separation significantly lowers the binding energy of excitons. As a result, a larger population of electrons can be efficiently trapped and stored in C_1_N_1_, thereby driving the two‐electron oxygen reduction toward H_2_O_2_.

**FIGURE 5:**
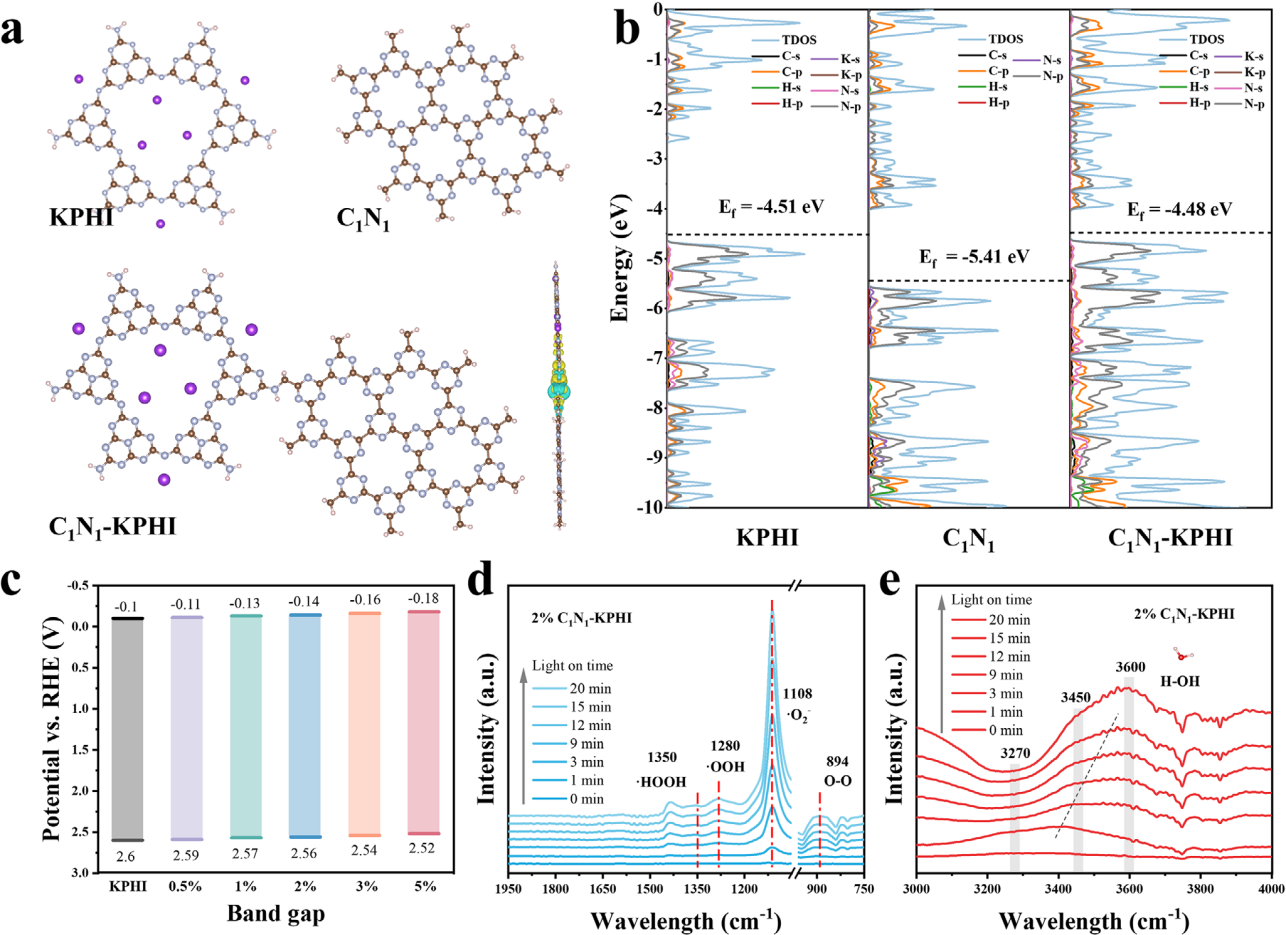
(a) DFT‐optimized structures for KPHI, C_1_N_1_, and the C_1_N_1_‐KPHI composite (with side‐view charge density difference on the right). (b) Projected density of states (PDOS) and corresponding Fermi levels of the as‐prepared samples. (c) Energy band positions of the samples derived from Mott–Schottky measurements. Time‐dependent in‐situ FTIR spectra of the 2%C_1_N_1_‐KPHI photocatalyst under light irradiation, showing (d) the evolution of signals from reactive intermediates and (e) the variation of surface‐adsorbed water signals.

To gain deeper insight into the interfacial charge transfer mechanism governing photocatalytic H_2_O_2_ evolution over the C_1_N_1_‐KPHI composite, density functional theory (DFT) calculations were performed. Initially, the structures of pristine KPHI, C_1_N_1_, and the composite C_1_N_1_‐KPHI were constructed and optimized, as shown in Figure 5a and Figure S17. Charge density difference analysis (Figure 5a and Figure S18) reveals significant charge redistribution at the interface upon composite formation, indicating strong electronic interaction between the two components. In Figure 5b, KPHI and C_1_N_1_ exhibit Fermi levels at −4.51 and −5.41 eV, respectively. The higher Fermi level of KPHI provides the thermodynamic driving force for electron transfer from KPHI to C_1_N_1_. Upon composite formation, the Fermi level shifts to −4.48 eV, which deviates from a simple average of the individual values, thus indicating that electrons flow from KPHI to C_1_N_1_ to establish a new electronic equilibrium. To further elucidate the electronic structure, density of states (DOS) and projected density of states (PDOS) analyses were conducted. Interestingly, DOS reveals that the electronic structure of the composite near the Fermi level closely resembles that of pristine KPHI rather than C_1_N_1_, indicating that KPHI serves as the primary contributor to the frontier electronic states. Meanwhile, PDOS further reveals strong orbital hybridization between the C‐p orbitals of C_1_N_1_ and the N‐p orbitals of KPHI at the interface (Figure 5b), providing a direct electronic coupling channel that facilitates interfacial charge transfer. In addition to these theoretical analyses, Mott–Schottky measurements were performed to investigate the band structure. As a carbonaceous material, C_1_N_1_ does not alter the intrinsic bandgap of KPHI, which is consistent with experimental observations in Figure 3a and Figure S19. Importantly, the band positions of the C_1_N_1_‐KPHI composite shift upward to a more negative potential compared with pristine KPHI in Figure 5c, further confirming the strong interfacial interaction and providing a thermodynamic advantage for the oxygen reduction reaction. To experimentally verify the reaction pathway, in‐situ FTIR spectroscopy was employed to monitor the intermediates during the photocatalytic process. The spectra clearly capture the characteristic signals of adsorbed O_2_ (894 cm^−^^1^) and key intermediates, including ∙O_2_^−^ (1108 cm^−^^1^), ∙OOH (1280 cm^−^^1^), and HOOH (1350 cm^−^^1^) [45, 46], confirming that the reaction proceeds via a superoxide‐mediated two‐electron reduction pathway from adsorbed O_2_ to H_2_O_2_. Notably, the in‐situ FTIR results also show a dynamic reconstruction of interfacial water molecules under light irradiation, with a gradual transition from bound water (3270 and 2450 cm^−1^) to free water (3600 cm^−1^) over increasing illumination time [47, 48]. This interfacial water reconstruction could facilitate proton transfer during the ORR, thereby accelerating the catalytic kinetics for H_2_O_2_ generation [49, 50]. Collectively, these findings consistently support a selective two‐electron ORR pathway promoted by the interfacial charge‐transfer architecture.

## 3 Conclusion

In summary, this work presented a facile approach to improve the photocatalytic reactivity of ionic carbon nitrides through the rational construction of an epitaxial heterojunction. The designed composite effectively extended light absorption to the visible range and promoted exciton dissociation and electron transfer kinetics at the material surface, likely driven by a built‐in electric field. The resultant optimal catalyst, designated as 2%C_1_N_1_‐KPHI, exhibited excellent performance for light‐driven H_2_O_2_ generation as a model reaction, achieving an AQY of 64% at 410 nm without relying on any cocatalysts and with glycerol as a relatively sluggish hole scavenger. Mechanistic insights from optical and photoelectrochemical analyses confirmed that the heterojunction structure facilitated superior charge separation and transport, directing electrons preferentially toward the C_1_N_1_ component and mitigating charge recombination. Electrochemical characterization results further substantiated these observations by indicating accelerated ORR kinetics via a two‐electron process. Notably, DFT calculations confirmed the thermodynamic driving force for electron transfer and interfacial orbital hybridization, while in situ FTIR spectroscopy identified key intermediates (∙O_2_^−^ and ∙OOH) and revealed interfacial water reconstruction that facilitates proton supply for ORR. More generally, this work underscores the significant potential of constructing heterojunctions based on ionic carbon nitrides as a promising pathway towards more active photocatalysts for a broad range of reactions.

## Author Contributions

**Chenyan Zhao**: validation, formal analysis. **Haijian Tong**: conceptualization, methodology, writing – review and editing, writing – original draft, validation, investigation, formal analysis, data curation, visualization. **Markus Antonietti**: conceptualization, methodology, writing – review and editing, resources, project administration, funding acquisition, supervision. **Christian Mark Pelicano**: conceptualization, methodology, writing – review and editing, supervision, resources, project administration.

## Conflicts of Interest

The authors declare no conflict of interest.

## Supporting information




**Supporting file**: advs77182‐sup‐0001‐SuppMat.docx

## Data Availability

The data that support the findings of this study are available from the corresponding author upon reasonable request.
