# Supplementary material for: Activating Ionic Carbon Nitrides via Epitaxial Dyadic Coupling for Efficient Solar‐Driven H2O2 Generation
Source: Adv Sci (Weinh). 2026 Aug 15:e77182. Online ahead of print. doi: 10.1002/advs.77182 (PMC13477258; doi:10.1002/advs.77182)
Supplement: Supplementary file 1 [file ADVS-9999-e77182-s001.docx]

Supporting Information

Activating Ionic Carbon Nitrides via Epitaxial Dyadic Coupling for Efficient Solar-Driven H_2_O_2_ Generation

Haijian Tong, ^1, *^ Chenyan Zhao, ^2^ Christian Mark Pelicano, ^1^ and Markus Antonietti ^1^

*^1^Department of Colloid Chemistry, Max Planck Institute of Colloids and Interfaces, Potsdam 14476, Germany*

*^2^State Key Laboratory of Precision and Intelligent Chemistry, University of Science and Technology of China, Hefei, Anhui 230026, China*

Corresponding author: haijian@ustc.edu.cn

**Experimental Section**

**1. Chemicals**

All chemicals and solvents used were purchased from different chemical suppliers (Thermo Scientific, Merck, Sigma-Aldrich) in high purity grade and were used as received. 5-Amino-1H-tetrazole monohydrate was dried in a vacuum oven before using.

**2. Catalyst Preparation**

2.1 Synthesis of KPHI

Potassium poly(heptazine imide) (KPHI) was synthesized following the methodology outlined in our prior publication. **^[1]^** The procedure involved taking 2.5 g of dried 5-Amino-1H-tetrazole monohydrate and 12.5 g of a KCl/LiCl eutectic mixture (in a 0.55/0.45 ratio) and placing them in a steel ball mill vessel. This mixture was then ground at an operational frequency of 25 Hz for 5 minutes. The resulting white powder was transferred to a porcelain crucible with a lid and heated in a furnace. The temperature of the furnace was gradually increased to 600 °C at a rate of 2.3 °C min^-1^ under a continuous flow of N_2_ gas (4 L/min) and held at this temperature for 4 h. After the heating process, the furnace was allowed to cool down naturally to room temperature. The product was then transferred from the crucible into a beaker with deionized H_2_O and stirred at room temperature overnight. Following this, the mixture was vacuum filtered, washed extensively with water through centrifugation, and dried in a vacuum oven at 60 °C overnight.

2.2 Synthesis of C_1_N_1_

The synthesis of C_1_N_1_ followed a similar procedure to KPHI. Specifically, 2.5g guanine was added to the KCl/LiCl eutectic salt, and the subsequent steps were identical to those for KPHI. The resulting catalysts were designated as C_1_N_1_.

2.3 Synthesis of *x%*C_1_N_1_-KPHI

The synthesis of *x%*C_1_N_1_-KPHI followed a similar procedure to KPHI, with a minor modification in the salt template. Specifically, 0.5-20 wt% (relative to the weight of 5-Amino-1H-tetrazole) of guanine was added to the KCl/LiCl eutectic salt mixture to prepare C_1_N_1_-KPHI with varying C_1_N_1_ content. The weight ratio of guanine to 5-Amino-1H-tetrazole was maintained between 0.5 and 20, and the subsequent steps were identical to those for KPHI. The resulting catalysts were designated as *x%*C_1_N_1_-KPHI, where *x* represents 0.5, 1, 2, 3, 5, 10 and 20, respectively.

**3. Characterizations**

X-ray powder diffraction (XRD) patterns were measured using a Rigaku Smart Lab (Japan, Cu K, 0.154 nm) at a generator voltage of 40 kV and a generator current of 50 mA, with a scanning speed of 2°/min from 5° to 80°. X-ray photoelectron spectroscopy (XPS) spectra were performed via Thermo Fisher Scientific ESCALAB 250Xi. Fourier transform infrared (FTIR) spectroscopy measurements were conducted using a Thermo Scientific Nicolet iD5 spectrometer with an attenuated total reflection sampling technique. Raman spectra were measured by laser [Raman spectroscopy](https://www.sciencedirect.com/topics/materials-science/raman-spectroscopy) (SuperLabRam II). Thermogravimetric Analysis (TGA) was performed using Shimadzu TGA-60H thermo-balance. This apparatus operated within a temperature range of approximately 25 to 900 °C, with a synthetic air flow rate of 50 mL/min and a heating rate of 10 °C/min in an alumina crucible. Inductively coupled plasma mass spectrometry (ICP-MS) was conducted using a PerkinElmer ICP-OES Optima 8000. Elemental combustion analysis (EA) was carried out with a vario MICRO cube CHNOS elemental analyzer from Elementar Analysensysteme GmbH. Nitrogen adsorption-desorption isotherms and pore size distributions were measured at 77 K with a Quantachrome Quadrasorb SI instrument. Prior to each measurement, the samples were degassed at 150 °C under a vacuum of 0.5 Torr for 15 hours. The specific surface area of each material was calculated from the adsorption branch data (P/P_0_ < 0.3) using the Brunauer-Emmett-Teller (BET) method. The sample morphologies were examined using a scanning electron microscope (SEM) (Zeiss LEO 1550-Gemini) with an energy-dispersive X-ray (EDX) detector (Oxford Instruments X-MAX). Transmission electron microscopy (TEM) images were captured using a JEOL JEM F200 and a double Cs corrected JEOL JEM-ARM 200F operating at 80 kV, equipped with a cold-field emission gun and a high-angle silicon drift EDX detector (Jeol JED 2300, with a solid angle up to 0.98 steradians and a detection area of 100 mm²). The optical properties and charge carrier behaviors were analyzed using UV-vis diffuse reflectance spectroscopy (UV-vis DRS, UV-2600, Shimadzu, Japan), electron paramagnetic resonance (EPR, Bruker EMXnano), steady-state photoluminescence (PL) with a Jasco FP-8300 fluorescence spectrometer at an excitation wavelength of 365 nm and time-resolved PL (TRPL), recorded on fluorescence lifetime spectrometer (FluoTime 250, PicoQuant) equipped with PDL 800-D picosecond pulsed diode laser drive. The average lifetime (τ_ave_) is calculated as follows:

τ_ave_ = (A_1_ τ_1_^2^ + A_2_ τ_2_^2^+ A_3_ τ_3_^2^) / (A_1_ τ_1_ + A_2_ τ_2_ +A_3_ τ_3_)	**(1)**

**4. Photoelectrochemical measurements**

All photoelectrochemical measurements were carried out in a three-electrode configuration, with a Pt wire and Ag/AgCl as counter and reference electrodes, respectively. The electrocatalysis experiments were conducted with a Gamry Interface 1010E potentiostat. To prepare the working electrode, F-doped Tin Oxide (FTO) glass (3 x 1 cm) substrates were cleaned sequentially with detergent, distilled H_2_O and ethanol for 15 min each to remove organic impurities. Half of the FTO area was protected with a Scotch tape. A catalyst ink was obtained by mixing 5 mg of photocatalyst powder, 0.5 mL of H_2_O and 20 μL of 5 wt.% Nafion by sonication for 30 min. Then, 50 μL of catalyst slurry was pipetted onto the FTO electrode and dried at 60 ℃ and further heated at 120 ℃ for 1 h to improve adhesion. All measured potentials were converted to reversible hydrogen electrode (RHE) according to the following equation (2):

*E_RHE_* = *E_Ag/AgCl_* + 0.059*pH + 0.197	**(2)**

4.1 Transient photocurrent response (TPR)

The photocurrent response was measured at 0 V versus reference electrode in 0.5 M aqueous Na_2_SO_4_ solution under white LED (100 mW cm^-2^) illumination using a Gamry Interface 1010E potentiostat.

4.2 Electrochemical impedance spectroscopy (EIS)

For EIS, the same electrodes were used as described above and the measurements were done in a frequency range of 10 kHz to 1 Hz. The data were fitted to a full semicircle using Z-View software.

4.3 Mott–Schottky measurements (MS)

M-S measurements were performed in a Biologic MPG-2 system at different frequencies using the same electrodes as described above.

**5. Radical quenching experiments**

A sample vial was filled with 5 mg 2%C_1_N_1_-KPHI powder in 2 mL water containing 10% glycerin along with a 10 mM sacrificial reagent: AgNO_3_ for e^-^, NaS_2_O_3_ for ·O_2_^-^, or t-butyl alcohol (TBA) for ·OH. The mixture was then purged with O_2_ gas for 1 min. The vial was irradiated under stirring using two 50W LED lamps (λ = 410 nm) for 1 h. The amount of generated H_2_O_2_ was quantified by spectrophotometry.

**6. EPR experiment**

The presence of ·O_2_⁻ in the reaction system was measured using spin trapping electron paramagnetic resonance (EPR). Typically, 5,5-Dimethyl-1-pyrroline N-oxide (DMPO) was employed as the spin-trapping reagent to detect radicals. The measurements were conducted in a H_2_O/MeOH mixture (1:4, 500 μL) containing 1 mg of the sample and 0.1 mmol DMPO, with a 410 nm LED as the light source.

**7. Evaluation of photocatalytic performance**

Initially, 5 mg of the catalyst was dispersed in 2 mL of an aqueous glycerin solution at a specified concentration, and O_2_ gas was bubbled through the mixture for 1 min. The reactor was then irradiated under stirring with two 50 W LED lamps (λ = 410 nm) for 1 h. After irradiation, the suspension was centrifuged at 1*10^4^ rpm for 10 minutes to separate the catalysts from the solution. The amount of generated H_2_O_2_ was quantified spectrophotometrically using the titanium oxalate method as previously described in the literature **^[1]^**. Specifically, a 10 g/L solution of K_2_[TiO(C_2_O_4_)_2_]·2H_2_O was prepared by dissolving the compound in 450 mL of water and 50 mL of H_2_SO_4_ to prevent complex precipitation. Then, 1.5 mL of this reagent was mixed with 0.5 mL of the supernatant from the photocatalytic experiment. The resulting solutions, diluted as necessary, were analyzed using a UV-vis spectrometer.

**8. Apparent quantum yield (AQY) estimation**

The AQY was measured using different monochromatic LEDs (365, 410, 465, 525 and 620 nm). The AQY was obtained by the following equation (3):

$\mathrm{AQY}\left( \% \right)=\frac{2*Rproduct*N_{A}*hc}{I*A* \lambda}* 100$**(3)**

where *R_product_* is the production rate of H_2_O_2_ molecules (mol s^−1^) after the 1 hour of photocatalytic reaction, *N_A_* is Avogadro constant (6.022 × 10^23^ mol^−1^), *h* is the Planck constant (6.626 × 10^−34^ J s^−1^) multiplied by *c* the speed of light (3 × 10^8^ m s^−1^) giving (1.98644586 × 10^−25^ J m), *A* is the irradiation area (cm^2^), *I* is the intensity of irradiation light (W cm^−2^), and *λ* is the wavelength of the monochromatic light (nm).

**9. H_2_O_2_ decomposition experiment**

To investigate the intrinsic H_2_O_2_ decomposition capability of the catalysts, the following procedure was adopted. First, 2 mg of catalyst was dispersed in 5 mL of 10% glycerol solution and illuminated for 1 hour under the same photocatalytic reaction conditions. After this pre-irradiation step, a certain amount of H_2_O_2_ was added to the suspension. The mixture was then subjected to another 1 hour of illumination. The H_2_O_2_ concentration before and after the second irradiation was measured to calculate the decomposition ratio.

**10. DFT calculation**

Density functional theory (DFT) calculations were performed using the Vienna Ab initio Simulation Package (VASP). The exchange–correlation interactions were described by the Perdew–Burke–Ernzerhof (PBE) functional within the generalized gradient approximation (GGA), and the projector augmented-wave (PAW) method was employed to account for core–valence electron interactions. Van der Waals interactions were corrected using Grimme’s D3 dispersion correction with Becke–Johnson damping. During geometry optimization, a plane-wave cutoff energy of 500 eV was applied, and the electronic self-consistent field convergence threshold was set to 1 × 10^-4^ eV. All atomic positions were fully relaxed while keeping the simulation cell fixed, until the maximum residual force was less than 0.10 eV·Å^-1^. Gaussian smearing with a width of 0.05 eV was used, and non-spin-polarized calculations were performed. Due to the use of large vacuum-separated finite-fragment models, Γ-point-only k-point sampling was adopted, which is sufficient for large supercell models with sufficient vacuum spacing.

To determine the preferred bonding configuration of the coupled structure, both single-bond and double-bond linkage modes between potassium-modified carbon nitride (K-CN) and the methyl-terminated triazine-based C–N fragment (MT-CN) were constructed and compared. The formation energies of the single- and double-bonded products were calculated as follows:

$\Delta E_{Form}= E_{K-MT}+ E_{H_{2}}- E_{K-CN}- E_{MT-CN}$**(4)**

$\Delta E_{Form}= E_{K=MT}+ {2E}_{H_{2}}- E_{K-CN}- E_{MT-CN}$**(5)**

where E_(K-MT) and E_(K=MT) are the total energies of the single-bonded and double-bonded coupled products, respectively; E_(K-CN) and E_(MT-CN) represent the total energies of potassium-modified carbon nitride and the methyl-terminated triazine-based C–N fragment, respectively; and E_(H_2_) is the total energy of an isolated hydrogen molecule calculated using the same computational settings. The calculated formation energy of the single-bonded product is 0.770 eV, which is significantly lower than that of the double-bonded product (2.970 eV). Therefore, the single-bonded configuration was selected for subsequent static self-consistent calculations, charge density analysis, and density of states (DOS) calculations.

Static self-consistent calculations were subsequently performed on the optimized structures to obtain total energies, charge densities, and density of states (DOS). The same PBE-D3 functional, plane-wave cutoff energy, smearing width, and Γ-point-only k-point sampling were used in these static calculations. Charge density files were generated for subsequent visualization and electronic structure analysis. The projected density of states (PDOS) was calculated with atom- and orbital-resolved projections using an energy grid of 2000 points to resolve fine electronic features near the Fermi level.

**11. Supplementary data**

**Figure** **S1.** (a) XRD patterns of KPHI and C_1_N_1_, (b) FTIR spectra of guanine and C_1_N_1_, (c) Raman spectra of C_1_N_1_, (d) TG curves of 5-Amino-tetrazole and guanine recorded under helium atmosphere (10 K min^-1^).

**Figure** **S2.** The (a-i) SEM micrographs of C_1_N_1_, KPHI and *x*%C_1_N_1_-KPHI.

**Figure** **S3.** High-angle annular dark-field scanning TEM (HAADF-STEM) images of C_1_N_1_ and their corresponding elemental mapping images.

**Figure** **S4.** HR-TEM images of (a) KPHI and (b-f) all *x*%C_1_N_1_-KPHI samples.

**Figure** **S5.** HR-TEM images of (a) KPHI and (b) 2%C_1_N_1_-KPHI with the corresponding TEM-EDS elemental mapping patterns, respectively.

**Table** **S1.** The relative ratios of C, N and H elementals in all samples determined by EA and the total amount of alkali metals in all catalysts via ICP.

| **Samples** | **C (%)** | **N (%)** | **H (%)** | **K (%)** | **Li (%)** |
| --- | --- | --- | --- | --- | --- |
| **C_1_N_1_** | 40.4 | 42.1 | 2.00 | 5.7 | 1.28 |
| **KPHI** | 26.9 | 45.0 | 2.1 | 10.5 | 0.35 |
| **0.5%C_1_N_1_-KPHI** | 27.4 | 46.2 | 2.1 | 10.2 | 0.40 |
| **1%C_1_N_1_-KPHI** | 27.4 | 45.4 | 2.1 | 10.8 | 0.35 |
| **2%C_1_N_1_-KPHI** | 27.3 | 44.7 | 2.0 | 11.3 | 0.31 |
| **3%C_1_N_1_-KPHI** | 27.6 | 45.5 | 2.2 | 11.3 | 0.29 |
| **5%C_1_N_1_-KPHI** | 27.5 | 45.1 | 2.1 | 10.8 | 0.32 |

**Figure S6.** (a) N_2_ adsorption/desorption isotherm at 77K, and (b) BJH pore size distribution from N_2_ adsorption branch at 77K of KPHI, C_1_N_1_ and all *x*%C_1_N_1_-KPHI.

**Table S2.** BET specific area pore volume and diameter size of all prepared samples.

| **Sample** | **S_BET_ m^2^ g^-1^** | **V_p_ cm^3^ g^-1^** | **D_p_ nm** |
| --- | --- | --- | --- |
| **C_1_N_1_** | 78.982 | 0.065 | 1.886 |
| **KPHI** | 36.852 | 0.093 | 1.886 |
| **0.5%C_1_N_1_-KPHI** | 39.295 | 0.089 | 1.898 |
| **1%C_1_N_1_-KPHI** | 38.811 | 0.089 | 1.885 |
| **2%C_1_N_1_-KPHI** | 49.982 | 0.083 | 1.877 |
| **5%C_1_N_1_-KPHI** | 50.422 | 0.092 | 1.857 |
| **20%C_1_N_1_-KPHI** | 68.378 | 0.090 | 1.862 |

**Figure S7.** (a) XPS full spectra, (b) high-resolution C1s and (c) N1s of C_1_N_1_.

**Figure S8.** (a) XPS full spectra, (b) high-resolution C1s and (c) N1s of KPHI and all *x*%C_1_N_1_-KPHI.

**Table S3.** N1s XPS quantitative results of KPHI and modified samples from deconvolution and N atom ratio.

| **Samples** | **C-N=C** | **N-C_3_** | **-NH_x_** | **N_2C_/N_3C_** |
| --- | --- | --- | --- | --- |
| **KPHI** | 0.65 | 0.15 | 0.15 | 4.3 |
| **0.5%C_1_N_1_-KPHI** | 0.64 | 0.15 | 0.14 | 4.3 |
| **1%C_1_N_1_-KPHI** | 0.62 | 0.2 | 0.13 | 3.1 |
| **2%C_1_N_1_-KPHI** | 0.62 | 0.2 | 0.13 | 3.1 |
| **3%C_1_N_1_-KPHI** | 0.63 | 0.17 | 0.14 | 3.7 |
| **5%C_1_N_1_-KPHI** | 0.63 | 0.16 | 0.14 | 3.9 |

**Figure S9.** EPR signals of KPHI and 2%C_1_N_1_-KPHI.

**Figure S10.** (a) HPLC chromatograms analysis spectra and (b) glycerol oxidation products for 2%C_1_N_1_-KPHI.

**Figure S11.** Decomposition of H_2_O_2_ over KPHI and 2%C_1_N_1_-KPHI under 1 h of light irradiation. (a) Photographs of the reaction solutions before and after the test. (b) Comparative degradation rates of the two catalysts.

**Figure S12.** UV-vis diffuse reflectance spectra of C_1_N_1_, KPHI, 2%C_1_N_1_-KPHI, and the corresponding physical mixture.

**Figure S13.** XRD patterns of 2%C_1_N_1_-KPHI before and after the recyclability tests for H_2_O_2_ production.

**Figure S14.** (a) UV-vis DRS spectra of KPHI and C_1_N_1_, (b) optical images of all prepared materials.

**Table S4.** Comparison of H_2_O_2_ production yields (μmol·g-1·h-1) and AQY (%) values with other carbon nitride photocatalysts synthesized by thermal condensation.

| **Photocatalyst** | **Modification** | **Conditions** | **H_2_O_2_ yields** | **AQY (%)** | **Ref.** |
| --- | --- | --- | --- | --- | --- |
| KPHI | — | LED_410nm_ | 5876 | 32.08  410 nm | **1** |
| *2%Ox*-KPHI | Oxamide induced | LED_410nm_ | 6772 | 40.72  410 nm | **1** |
| *2.5%*AC-KPHI | NH_4_Cl induced | LED_410nm_ | 4920 | 48.9  420nm | **2** |
| Bulk g-C_3_N_4_ | — | Xe-lamp  (λ>420nm) | 125 | 7  420nm | **3** |
| Nv-g-C_3_N_4_ nanosheets | N vacancy | Xe-lamp  (λ>420nm) | 1768 | 10.5  420nm | **4** |
| Sb-doped  g-C_3_N_4_ | Sb doping | LED_420nm_ | 3480 | 17.6  420 nm | **5** |
| WSe_2_/g-C_3_N_4_ | Z-scheme heterojunction | Xe-lamp  (λ > 420nm) | 40.62 | 7.18  420 nm | **6** |
| Au/g-C_3_N_4_ | Au Co-catalyst | Xe-lamp  (λ > 420nm) | 82.5 | 3.63  400 nm | **7** |
| g-C_3_N_4_ with hole defects | Hole defects | AM1.5  (λ > 420 nm) | 5.81 | 10.2  420 nm | **8** |
| HTCN | H/T junction | visible light  (400 < λ < 800 nm) | 22825.5 | 21.5  420 nm | **9** |
| PDI/CNA | D-A structure | LED  (400 ≤ λ ≤ 760 nm ) | 1605.32 | 27.18  400 nm | **10** |
| Alkali metal-  doped Nv-g-C_3_N_4_ | N vacancy,  K, Na doping | 300 W Xe-lamp  (λ > 400 nm) | 3080 | 6.8  400 nm | **11** |
| Nv–CN–CN | N vacancy,  –C≡N group | 300 W Xe-lamp  (λ ≥ 420 nm) | 3093 | 22.1  420 nm | **12** |
| ***2%*C_1_N_1_-KPHI** | Heterojunction | LED_410nm_ | 13172 | **63.94**  **410 nm** | **This**  **work** |

**Figure S15.** Photocatalytic H_2_O_2_ production under different testing conditions.

**Figure S16.** EPR spectra of DMPO-⋅OH over 2%C_1_N_1_-KPHI.

**Figure S17.** Optimized geometric configurations of the C_1_N_1_-KPHI composite with different formation energies: (a) the more stable structure with an energy of 0.77 eV (final optimized structure), and (b) a higher-energy configuration at 2.97 eV.

**Figure S18.** Charge density difference map of the most stable C_1_N_1_-KPHI configuration, showing interfacial charge redistribution upon composite formation.

**Figure S19.** Mott–Schottky (M-S) plots of pristine KPHI and *x*%C_1_N_1_-KPHI with varying C_1_N_1_ contents, revealing the semiconductor type and flat-band potentials of the as-prepared samples.

**References**

[1] H. Tong, J. Odutola, J. Song, L. Peng, N. Tkachenko, M. Antonietti, C. M. Pelicano, Boosting the Quantum Efficiency of Ionic Carbon Nitrides in Photocatalytic H_2_O_2_ Evolution via Controllable n → π * Electronic Transition Activation, *Adv. Mater*., **2024**, 36, 2412753.

[2] J. Bharti, J. Odutola, Z. Hajiahmadi, K. Nolkemper, Z. Tian, H. Tong, V. Shvalagin, T. D. Kühne, T.-P. Ruoko, C. M. Pelicano, NH_3_‑Guided Low‑Temperature Nanostructural Refinement Boosts Visible‑Light‑Driven H_2_O_2_ Synthesis in Ionic Carbon Nitrides, *Adv. Mater.*, **2026**, 38, e10585.

[3] Y. Shiraishi, S. Kanazawa, Y. Sugano, D. Tsukamoto, H. Sakamoto, S. Ichikawa, T. Hirai, Highly Selective Production of Hydrogen Peroxide on Graphitic Carbon Nitride (g‑C_3_N_4_) Photocatalyst Activated by Visible Light, *ACS Catal.*, **2014**, 4, 774–780.

[4] H. Zhao, C. Shi, Q. Li, X. Wang, G. Zeng, S. Ye, B. Jiang, J. Liu, Nitrogen Vacancy-Rich Porous Carbon Nitride Nanosheets for Efficient Photocatalytic H_2_O_2_ Production, *Mater. Today Energy*, **2022**, 24, 100926.

[5] Z. Teng, Q. Zhang, H. Yang, K. Kato, W. Yang, Y.-R. Lu, S. Liu, C. Wang, A. Yamakata, C. Su, B. Liu, T. Ohno, Atomically Dspersed Antimony on Carbon Nitride for the Artificial Photosynthesis of Hydrogen Peroxide, *Nat. Catal.*, **2021**, 4, 374–384.

[6] W. Wang, W. Gu, G. Li, H. Xie, P. K. Wong, T. An, Few-Layered Tungsten Selenide as a Co-Catalyst for Visible-Light-Driven Photocatalytic Production of Hydrogen Peroxide for Bacterial Inactivation, *Environ. Sci.: Nano*, **2020**, 7, 3877–3887.

[7] X. Chang, J. Yang, D. Han, B. Zhang, X. Xiang, J. He, Enhancing Light‑Driven Production of Hydrogen Peroxide by Anchoring Au onto C_3_N_4_ Catalysts, *Catalysts*, **2018**, 8, 147.

[8] L. Shi, L. Yang, W. Zhou, Y. Liu, L. Yin, X. Hai, H. Song, J. Ye, Photoassisted Construction of Holey Defective g‑C_3_N_4_ Photocatalysts for Efficient Visible‑Light‑Driven H_2_O_2_ Production, *Small*, **2018**, 14, 1703142.

[9] Y. Zhang, Q. Cao, A. Meng, X. Wu, Y. Xiao, C. Su, Q. Zhang, Molecular Heptazine‑Triazine Junction over Carbon Nitride Frameworks for Artificial Photosynthesis of Hydrogen Peroxide, *Adv. Mater.*, **2023**, 35, 2306831.

[10] J. Hu, C. Chen, H. Yang, F. Yang, J. Qu, X. Yang, W. Sun, L. Dai, C. M. Li, Tailoring Well-Ordered, Highly Crystalline Carbon Nitride Nanoarrays via Molecular Engineering for Efficient Photosynthesis of H_2_O_2_, *Appl. Catal. B Environ. Energy*, **2022**, 317, 121723.

[11] Q. Bai, Y. Huang, Z. Chen, Y. Pan, X. Zhang, Q. Long, Q. Yang, T. Wu, T-Z. Xie, M. Wang, H. Luo, C. Hu, P. Wang, Z. Zhang, Terpyridine-Based Metallo-Cuboctahedron Nanomaterials for Efficient Photocatalytic Degradation of Persistent Organic Pollutants, *Nano Res.*, **2024**, 17, 6833–6840.

[12] X. Zhang, P. Ma, C. Wang, L. Gan, X. Chen, P. Zhang, Y. Wang, H. Li, L. Wang, X. Zhou, K. Zheng, Unraveling the Dual Defect Sites in Graphite Carbon Nitride for Ultra‑High Photocatalytic H_2_O_2_ Evolution, *Energy Environ. Sci.*, **2022**, 15, 830–842.
